# Clinical impact of follow‐up imaging on mortality in Korean breast cancer patients: A national cohort study

**DOI:** 10.1002/cam4.3873

**Published:** 2021-09-01

**Authors:** So‐Youn Jung, Young Ae Kim, Dong‐Eun Lee, Jungnam Joo, Joung Hwan Back, Sun‐Young Kong, Eun Sook Lee

**Affiliations:** ^1^ Department of Surgery National Cancer Center Goyang Korea; ^2^ National Cancer Center Graduate School of Cancer Science and Policy Goyang Korea; ^3^ Cancer Policy Branch National Cancer Control Institute National Cancer Center Goyang Korea; ^4^ Biostatistics Collaboration Team Research Core Center Research Institute National Cancer Center Goyang Korea; ^5^ Office of Biostatistics Research National Heart, Lung and Blood Institute National Institutes of Health Bethesda MD USA; ^6^ Health Insurance Policy Research Institute National Health Insurance Service Wonju South Korea; ^7^ Department of Laboratory Medicine National Cancer Center Goyang Korea; ^8^ Division of Translational Science Research Institute National Cancer Center Goyang Korea

**Keywords:** breast cancer, follow‐up, image, surgery, surveillance, survivors

## Abstract

**Background:**

As the incidence of breast cancer has increased and the survival rate has improved, supporting the optimal follow‐up strategy has become an important issue. This study aimed to evaluate follow‐up imaging usage after breast cancer surgery and the implications on mortality in Korea.

**Methods:**

This study included 96,575 breast cancer patients diagnosed during 2002–2010 and registered in the Korea Central Cancer Registry, Statistics Korea, and Korean National Health Insurance Service. We evaluated the frequency of breast imaging (mammography and breast MRI) and systemic imaging for evaluating the presence of distant metastasis (chest CT, bone scan, and PET‐CT), and performed analyses to determine if they had an effect on mortality.

**Results:**

The median follow‐up period was 72.9 months (range: 12.0–133.3) and 7.5% of the patients died. Among all patients, 54.7%, 16.2%, 45.6%, and 8.5% received 3 or more mammograms, chest CTs, bone scans, and PET‐CTs within 3 years after surgery, respectively. Among patients who developed recurrence after 3 or more years, a comparison of overall mortality and breast‐cancer specific mortality according to the frequency of imaging by modality (<3 vs. ≥3) showed that only mammography had significantly reduced mortality (hazard ratio [HR]: 0.72, 95% CI: 0.61–0.84, *p *< 0.0001; HR: 0.72, 95% CI: 0.61–0.84; *p *< 0.0001).

**Conclusions:**

This study showed that only frequent mammography reduced mortality and frequent imaging follow‐up with other modalities did not when compared to less frequent imaging. This finding provides supportive evidence that clinicians need to adhere to the current guidelines for surveillance after breast cancer surgery.

## Graphical abstract

This study aimed to evaluate follow‐up imaging usage and the clinical implications after breast cancer surgery using Korea national cohort. We found that only frequent mammography reduced mortality and other imaging modalities did not. Therefore, clinicians need to adhere to the current guidelines for surveillance after breast cancer surgery.

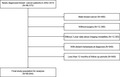

## 1 INTRODUCTION

As the incidence of breast cancer has increased and the survival rate has improved,^1^, ^2^ determining the ideal follow‐up strategy has become an important issue. Follow‐up in breast cancer patients is aimed at the detection of recurrence, metastasis, or new primary cancers; evaluation of treatment‐related long‐term or late effects, adherence to the recommended therapy and screening; and psychosocial and decision‐making support.^3^, ^4^

Current guidelines recommend regular follow‐up with history taking, physical examination, and annual mammography to detect new primary cancers, recurrence, and treatment‐related adverse effects.^5^, ^6^ In contrast, they do not recommend regular systemic imaging such as chest computed tomography (CT), bone scan, and positron emission tomography (PET)‐CT for the follow‐up of asymptomatic breast cancer patients. These recommendations are based on results from prior studies which established that early diagnosis of distant metastasis provides no additional advantage for survival or health‐related quality of life (QoL).^7^, ^8^

Despite these guidelines, the patient fear of recurrence and clinician inclination for early detection of disease recurrence result in frequent usage of systemic imaging.^9^, ^10^ In a previous survey of medical and surgical breast oncologists conducted by the Korean Breast Cancer Society, 50% of respondents indicated that they perform follow‐up chest CT more than once a year for the first 5 years and PET‐CT more than once a year for the first 3 years.^11^

This study aimed to evaluate the recent clinical usage of follow‐up imaging by frequency and modality after curative treatment among Korean breast cancer patients and their implications on mortality. Towards this goal, we analyzed the combined data of the Korea Central Cancer Registry (KCCR), Statistics Korea, and Korean National Health Insurance Service (KNHIS) (Big Data‐Based Guideline for Work‐up and Interval after Surgery in Breast Cancer Patients: BIG‐WISE Study).

## 2 METHODS

### 2.1 Study design and population

This BIG‐WISE study was approved by the Institutional Review Board of the National Cancer Center, Korea (NCC 2016‐0209), and the requirement for informed consent was waived because of the use of de‐identified data. The subjects were Korean female breast cancer patients (with International Classification of Diseases, 10th revision [ICD‐10] code^12^) diagnosed between 2002 and 2010 and registered in the KCCR, Statistics Korea, and KNHIS.

**FIGURE 1:**
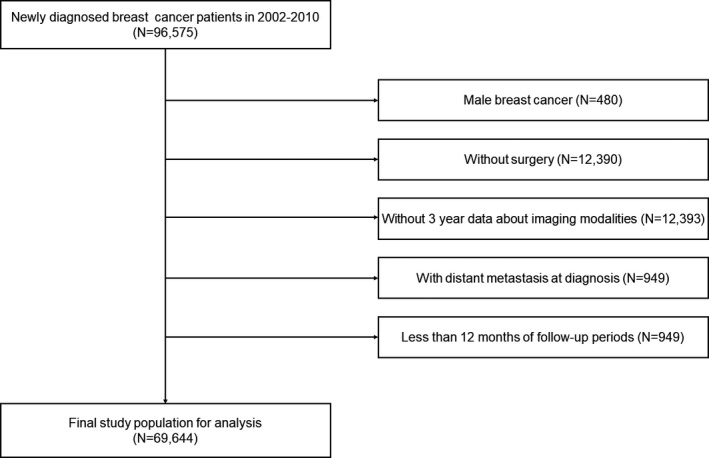
Study population from National Cohort

Of the 96,575 breast cancer patients initially identified, we excluded 27,031 patients who were male (*n* = 480), did not undergo breast cancer surgery (*n* = 12,390), did not have 3 years of data on imaging in the KNHIS (*n* = 12,393), had distant metastasis at diagnosis (*n* = 949), or had less than 12 months of follow‐up (*n* = 816) (Figure 1). Finally, 69,544 breast cancer patients were included in the analysis. Age at diagnosis was classified as <30, 30–39, 40–49, 50–59, 60–69, and ≥70 years. Comorbidities were evaluated using the Charlson Comorbidity Index (CCI) and categorized as 0, 1, and ≥2. Stage at diagnosis, the data on which became available in the KCCR starting in 2005, was classified as local, regional, and missing/unknown following the Surveillance, Epidemiology, and End Results staging system.^13^

### 2.2 Data sources

For this BIG‐WISE study, we established a merged database from three national cohorts: KCCR, KNHIS, and Statistics Korea. KCCR is a population‐based national cancer registry that includes information on more than 98% of patients with newly diagnosed cancer in Korea.^14^, ^15^ The KNHIS is the single insurer of the Korean public health system, and tracks medical information including cancer treatment status (chemotherapy, radiotherapy, and endocrine therapy) and imaging studies.^16^ Mortality data were obtained from Statistics Korea.^17^

### 2.3 Imaging modalities

As follow‐up imaging modalities after surgery, breast imaging (mammography and breast MRI), systemic scans to assess the presence of distant metastasis (chest X‐ray, chest CT, bone scan, and PET‐CT) and treatment‐related imaging (DEXA) were evaluated. We investigated the date at which each follow‐up modality was performed and the number of studies performed for each modality.

To assess the effect of follow‐up imaging on clinical outcomes, patients were divided into two groups according to the number of studies performed for each modality. A cut‐off value of 3 was set based on annual check for 3 years. Univariate and multivariate analyses were performed comparing overall and breast cancer‐related mortality between the divided groups for each imaging modality. An additional analysis was performed in patients with recurrence after 3 or more years to determine whether the frequent follow‐up imaging during 3 years after curative treatment can improve clinical outcomes in recurrent patients.

### 2.4 Assessment of recurrence and death

Mortality data were obtained from Statistics Korea with the date of the last follow‐up as December 31, 2011.^17^ The cause of death was recorded and classified according to ICD‐10 code.^12^ Because KCCR and KNHIS did not have the exact medical information on the date or site of recurrence, we defined recurrence as the presence of new breast cancer surgery, chemotherapy, or radiotherapy, or a change of hormonal therapy, excluding switching and extended therapy, after completion of curative treatments.

### 2.5 Statistical analysis

Baseline characteristics and imaging modality after surgery were expressed as frequencies in percent. Between‐group comparisons of patient characteristics at diagnosis and treatment status (adjuvant chemotherapy, adjuvant radiotherapy, adjuvant hormonal therapy) were performed using the chi‐squared test or Fisher's exact test, as appropriate. Cox proportional model was used to evaluate the association between patient survival and the frequency of imaging follow‐up for each modality after surgery. Age at diagnosis, treatment status (chemotherapy, radiotherapy, adjuvant hormonal therapy), and CCI without cancer were adjusted for in the multivariable Cox proportional hazard model. All statistical analyses were performed using SAS 9.4 (SAS Institute Inc.), and a *p* value <0.05 was considered statistically significant.

## 3 RESULTS

### 3.1 Patient characteristics

Baseline characteristics for 69,544 breast cancer patients are summarized in Table 1. The most common age at diagnosis was 40–49 years (28,383/69,544; 40.8%), followed by 50–59 years (17,358/69,544; 25.0%). In total, 36.8% (25,583/69,544) and 25.3% (17,617/69,544) of the patients had localized disease and regional metastasis, respectively, and 14.4% patients (10,032/69,544) had a CCI score of ≥3. Overall, 76.3% (53,035/69,544) underwent adjuvant chemotherapy; 61.1% (42,504/69,544), adjuvant radiotherapy; and 69.8% (48,511/69,544), adjuvant hormonal therapy.

**TABLE 1:** Baseline patient characteristics

		*N*	%
Total		69,544	100.0
Age at diagnosis, years	<30	1157	1.7
	30–39	10,905	15.7
	40–49	28,383	40.8
	50–59	17,358	25.0
	60–69	8630	12.4
	≥70	3111	4.5
Stage at diagnosis^a^	Localized	25,583	36.8
	Regional	17,617	25.3
	Unknown or missing	26,344	37.9
Year of diagnosis	2002	6237	9.0
	2003	6969	10.0
	2004	7388	10.6
	2005	8049	11.6
	2006	9069	13.0
	2007	9947	14.3
	2008	10,697	15.4
	2009	11,188	16.1
Charlson comorbidity index^b^	0	22,333	32.1
	1	23,892	34.4
	2	13,287	19.1
	≥3	10,032	14.4
Adjuvant chemotherapy	No	16,509	23.7
	Yes	53,035	76.3
Adjuvant radiotherapy	No	27,040	38.9
	Yes	42,504	61.1
Adjuvant hormonal therapy	No	21,033	30.2
	Yes	48,511	69.8
Recurrence	No	57,868	83.2
	Yes	11,676	16.8
Death	No	64,303	92.5
	Yes	5241	7.5
Cause of death	Breast cancer	4357	83.1
	Other cancer	350	6.7
	Other cause	502	9.6
	Missing	32	0.6

^a^ Stage was evaluated since 2005.

^b^ Except cancer.

The median follow‐up period was 72.9 months (range: 12.0–133.3), and 11,676 patients (16.8%) experienced recurrence and 5241 (7.5%) died, 83.1% of whom died from breast cancer.

### 3.2 Imaging follow‐up

After completion of surgery, 93.1% (64,754/69,544) of survivors underwent mammography; 6.8% (4737/69,544), breast MRI; 94.5% (65,713/69,544), chest radiography; 71.3% (49,588/69,544), chest CT; 84.5% (58,746/69,544), bone scan; 67.1% (46,680/69,544), PET‐CT; and 53.8% (37,389/69,544), DEXA. In total, 97.2% (67,587/69,544) underwent CT, bone scan, or PET at least once (Table 2). Within 3 years of breast cancer surgery, ≥1 and ≥3 mammography examinations were performed in 89.6% (62,288/69,544) and 54.7% (27,353/69,544, 39.3%, 3–4; 10,669/69,544, 15.4%, ≥5); chest radiography in 88.6% (61,584/69,544) and 66.5% (46,212/69,544); chest CT in 40.3% (28,007/69,544) and 16.2% (11,274/69,544); bone scan in 78.7% (54,740/69,544) and 45.6% (31,719/69,544); and PET‐CT in 40.3% (28,052/69,544) and 8.5% (5927/69,544).

**TABLE 2:** Follow‐up frequency and modality after surgery by time period

			Total		≤6 months		6 −12 months		12–24 months		24–36 months		>36 months		≤36 months	
			*N*	%	*N*	%	*N*	%	*N*	%	*N*	%	*N*	%	*N*	%
Local work‐up																
Mammography	No		4790	6.9	53,995	77.6	30,735	44.2	15,735	22.6	17,181	25.2	14,666	22.4	7256	10.4
	Yes	All	64,754	93.1	15,549	22.4	38,809	55.8	53,809	77.4	51,093	74.8	50,760	77.6	62,288	89.6
		1 or 2	9309	13.4	15,498	22.3	38,491	55.4	50,897	73.2	49,019	71.8	21,608	33.1	24,262	34.9
		3 or 4	15,342	22.0	51	0.1	302	0.4	2795	4.0	2001	2.9	15,457	23.6	27,357	39.3
		≥5	40,103	57.7	0	0.0	16	0.0	117	0.2	73	0.1	13,695	20.9	10,669	15.4
Breast MRI^a^	No		64,807	93.2	69,082	99.3	68,978	99.2	68,287	98.2	66,945	98.1	62,612	95.7	67,267	96.7
	Yes	All	4737	6.8	462	0.7	566	0.8	1257	1.8	1329	1.9	2814	4.3	2277	3.3
		1	3138	4.5	455	0.7	546	0.8	1121	1.6	1262	1.8	2230	3.4	1578	2.3
		2	828	1.2	7	0.0	19	0.0	125	0.2	64	0.1	378	0.6	406	0.6
		≥3	771	1.1	0	0.0	1	0.0	11	0.0	3	0	206	0.3	293	0.4
Systemic work‐up																
Chest radiography	No		3831	5.5	28,146	40.5	24,822	35.7	14,820	21.3	17,231	25.2	15,397	23.5	7960	11.5
	Yes	All	65,713	94.5	41,398	59.5	44,722	64.3	54,724	78.7	51,043	74.8	50,029	76.5	61,584	88.6
		1 or 2	6147	8.8	24,749	35.6	39,345	56.6	45,721	65.8	44,286	64.9	17,838	27.3	15,372	22.1
		3 or 4	7718	11.1	6905	9.9	4055	5.8	6332	9.1	4124	6	13,325	20.4	23,720	34.1
		≥5	51,848	74.6	9744	14.0	1322	1.9	2671	3.8	2633	3.9	18,866	28.8	22,492	32.4
Chest CT	No		19,956	28.7	40,746	58.6	53,316	76.7	51,977	74.7	49,381	72.3	40,485	61.9	41,537	59.7
	Yes	All	49,588	71.3	28,798	41.4	16,228	23.3	17,567	25.3	18,893	27.7	24,941	38.1	28,007	40.3
		1 or 2	25,228	36.3	28,132	40.5	15,605	22.4	15,597	22.4	16,653	24.4	13,582	20.8	16,733	24.1
		3 or 4	8199	11.8	652	0.9	606	0.9	1528	2.3	1554	2.3	5412	8.2	7204	10.4
		≥5	16,161	23.2	14	0.0	17	0.0	442	0.6	686	1	5947	9.1	4070	5.8
Bone scan	No		10,798	15.5	53,335	76.7	36,224	52.1	23,116	33.2	24,089	35.3	20,194	30.9	14,804	21.3
	Yes	All	58,746	84.5	16,209	23.3	33,320	47.9	46,428	66.8	44,185	64.7	45,232	69.1	54,740	78.7
		1 or 2	11,375	16.4	16,174	23.2	33,246	47.8	44,618	64.2	42,633	62.4	21,633	33.0	23,021	33.1
		3 or 4	14,116	20.3	35	0.1	74	0.1	1,770	2.5	1,467	2.2	12,430	19.0	22,616	32.5
		≥5	33,255	47.8	0	0.0	0	0.0	40	0.1	89	0.1	11,169	17.1	9103	13.1
PET‐CT^b^	No		22,864	32.9	65,598	94.3	60,426	86.9	52,814	75.9	50,103	73.4	31,999	48.9	41,492	59.7
	Yes	All	46,680	67.1	3946	5.7	9118	13.1	16,730	24.1	18,171	26.6	33,427	51.1	28,052	40.3
		1 or 2	28,578	41.1	3809	5.5	9027	13.0	16,146	23.2	17,765	26	28,137	43.0	22,125	31.8
		3 or 4	13,076	18.8	129	0.2	87	0.1	546	0.8	359	0.5	3909	6.0	5010	7.2
		≥5	5026	7.2	8	0.0	4	0.0	38	0.1	47	0.1	1381	2.1	917	1.3
DEXA	No		32,155	46.2	65,725	94.5	61,705	88.7	54,248	78.0	51,315	75.2	38,444	58.8	45,052	64.8
	Yes	All	37,389	53.8	3819	5.5	7839	11.3	15,296	22.0	16,959	24.8	26,982	41.2	24,492	35.2
		1	10,759	15.5	3747	5.4	7718	11.1	14,311	20.6	16,167	23.7	12,589	19.2	11,743	16.9
		2	8769	12.6	71	0.1	121	0.2	940	1.3	783	1.1	7380	11.3	9288	13.3
		≥3	17,861	25.7	1	0.0	0	0.0	45	0.1	9	0	7013	10.7	3461	5.0
PET‐CT, CT, or bone scan	No		1957	2.8	31,300	45.0	26,727	38.4	15,163	21.8	14,504	21.2	11,072	16.9	6765	9.7
	Yes	All	67,587	97.2	38,244	55.0	42,817	61.6	54,381	78.2	53,770	78.8	54,354	83.1	62,779	90.3
		1 or 2	6802	9.8	33,243	47.8	36,183	52.0	36,612	52.7	35,720	52.4	15,614	23.9	14,082	20.2
		3 or 4	7473	10.7	4288	6.2	5672	8.2	13,369	19.2	13,532	19.8	11,636	17.8	18,964	27.3
		≥5	53,312	76.7	713	1.0	962	1.4	4400	6.3	4518	6.6	27,104	41.4	29,733	42.8

^a^ Evaluated since 2005.

^b^ Since 2006.

Young women aged <30 years underwent less mammography (576/1157, 49.8%, *p *< 0.001) and more breast MRI (73/1157, 6.3%, *p *< 0.001, Table 3). Old women aged ≥70 years had less imaging work‐up (mammography: 37.6% (1170/3111), breast MRI: 1.3% (39/3111), chest radiography: 48.5% (1509/3111), chest CT: 11.0% (343/3111), bone scan: 27.5% (856/3111), and PET‐CT: 5.9% (183/3111), Table 3). Patients with local disease underwent more mammography (14,987/25,583, 58.6%) and breast MRI (1281/25,583, 5.0%). Systemic imaging work‐ups were performed in patients with regional disease, those who underwent chemotherapy, and those who underwent radiotherapy (Table 3). Patients who underwent adjuvant hormonal therapy underwent more bone scan (46.9% (22,729/48,511) vs. 42.7% (8990/21,033), *p *< 0.001) and less chest CT (15.6% (7589/48,511) vs. 17.5% (3685/21,033), *p *< 0.001) and PET‐CT (7.9% (3826/48,511) vs. 10.0% (2101/21,033), *p *< 0.001).

**TABLE 3:** Comparison of follow‐up imaging frequency and modality within 3 years after treatment based on clinical characteristics

		Total	Age at diagnosis (years)													Stage at diagnosis						
			<30		30–39		40–49		50–59		60–69		≥70		*p*‐value	Localized		Regional		Unstaged or missing		*p*‐value
			*N*	%	*N*	%	*N*	%	*N*	%	*N*	%	*N*	%		*N*	%	*N*	%	*N*	%	
Follow‐up imaging		69,544	1157	100	10,905	100	28,383	100	17,358	100	8630	100	3111	100		25,583	100	17,617	100	26,344	100	
Mammography^a^	<3	31,518	581	50.2	4930	45.2	12,187	42.9	7705	44.4	4174	48.4	1941	62.4	<0.0001	10,596	41.4	8089	45.9	12,833	48.7	<0.0001
	≥3	38,026	576	49.8	5975	54.8	16,196	57.1	9653	55.6	4456	51.6	1170	37.6		14,987	58.6	9528	54.1	13,511	51.3	
Breast MRI^b^	No	67,267	1084	93.7	10,483	96.1	27,394	96.5	16,809	96.8	8425	97.6	3072	98.7	<0.0001	24,302	95.0	16,899	95.9	26,066	98.9	<0.0001
	Yes	2277	73	6.3	422	3.9	989	3.5	549	3.2	205	2.4	39	1.3		1281	5.0	718	4.1	278	1.1	
Chest radiography^a^	<3	23,332	367	31.7	3365	30.9	9090	32.0	5698	32.8	3210	37.2	1602	51.5	<0.0001	9683	37.8	5490	31.2	8159	31.0	<0.0001
	≥3	46,212	790	68.3	7540	69.1	19,293	68.0	11,660	67.2	5420	62.8	1509	48.5		15,900	62.2	12,127	68.8	18,185	69.0	
Chest CT^a^	<3	58,270	963	83.2	8964	82.2	23,856	84.1	14,488	83.5	7231	83.8	2768	89.0	<0.0001	21,499	84.0	13,107	74.4	23,664	89.8	<0.0001
	≥3	11,274	194	16.8	1941	17.8	4527	15.9	2870	16.5	1399	16.2	343	11.0		4084	16.0	4510	25.6	2680	10.2	
Bone scan^a^	<3	37,825	636	55.0	5755	52.8	15,002	52.9	9317	53.7	4860	56.3	2255	72.5	<0.0001	14,961	58.5	9248	52.5	13,616	51.7	<0.0001
	≥3	31,719	521	45.0	5150	47.2	13,381	47.1	8041	46.3	3770	43.7	856	27.5		10,622	41.5	8369	47.5	12,728	48.3	
PET‐CT^a^	<3	63,617	1040	89.9	9918	90.9	26,067	91.8	15,756	90.8	7908	91.6	2928	94.1	<0.0001	23,027	90.0	14,961	84.9	25,629	97.3	<0.0001
	≥3	5927	117	10.1	987	9.1	2316	8.2	1602	9.2	722	8.4	183	5.9		2556	10.0	2656	15.1	715	2.7	
DEXA^a^	Yes	45,052	935	80.8	8314	76.2	18,970	66.8	9874	56.9	4947	57.3	2012	64.7	<0.0001	14,271	55.8	9478	53.8	21,303	80.9	<0.0001
	No	24,492	222	19.2	2591	23.8	9413	33.2	7484	43.1	3683	42.7	1099	35.3		11,312	44.2	8139	46.2	5041	19.1	
CT or PET‐CT or Bone scan^a^	<3	20,847	391	33.8	3243	29.7	8201	28.9	4934	28.4	2584	29.9	1494	48.0	<0.0001	8075	31.6	3555	20.2	9217	35.0	<0.0001
	≥3	48,697	766	66.2	7662	70.3	20,182	71.1	12,424	71.6	6046	70.1	1617	52.0		17,508	68.4	14,062	79.8	17,127	65.0	

		Adjuvant chemotherapy					Adjuvant radiotherapy					Adjuvant hormonal therapy				
		No		Yes		*p*‐value	No		Yes		*p*‐value	No		Yes		*p*‐value
		*N*	%	*N*	%		*N*	%	*N*	%		*N*	%	*N*	%	
Follow‐up imaging		16,509	100	53,035	100		27,040	100	42,504	100		21,033	100	48,511	100	
Mammography^a^	<3	7548	45.7	23,970	45.2	0.24	13,888	51.4	17,630	41.5	<0.0001	10,600	50.4	20,918	43.1	<0.0001
	≥3	8961	54.3	29,065	54.8		13,152	48.6	24,874	58.5		10,433	49.6	27,593	56.9	
Breast MRI^b^	No	15,999	96.9	51,268	96.7	0.13	26,680	98.7	40,587	95.5	<0.0001	20,202	96.0	47,065	97.0	<0.0001
	Yes	510	3.1	1767	3.3		360	1.3	1917	4.5		831	4.0	1446	3.0	
Chest radiography^a^	<3	6597	40.0	16,735	31.6	<0.0001	10,487	38.8	12,845	30.2	<0.0001	7675	36.5	15,657	32.3	<0.0001
	≥3	9912	60.0	36,300	68.4		16,553	61.2	29,659	69.8		13,358	63.5	32,854	67.7	
Chest CT^a^	<3	15,096	91.4	43,174	81.4	<0.0001	24,127	89.2	34,143	80.3	<0.0001	17,348	82.5	40,922	84.4	<0.0001
	≥3	1413	8.6	9861	18.6		2913	10.8	8361	19.7		3685	17.5	7589	15.6	
Bone scan^a^	<3	11,167	67.6	26,658	50.3	<0.0001	15,805	58.5	22,020	51.8	<0.0001	12,043	57.3	25,782	53.1	<0.0001
	≥3	5342	32.4	26,377	49.7		11,235	41.5	20,484	48.2		8990	42.7	22,729	46.9	
PET‐CT^a^	<3	15,816	95.8	47,801	90.1	<0.0001	25,307	93.6	38,310	90.1	<0.0001	18,932	90.0	44,685	92.1	<0.0001
	≥3	693	4.2	5234	9.9		1733	6.4	4194	9.9		2101	10.0	3826	7.9	
DEXA^a^	No	9821	59.5	35,231	66.4	<0.0001	18,822	69.6	26,230	61.7	<0.0001	15,482	73.6	29,570	61.0	<0.0001
	Yes	6688	40.5	17,804	33.6		8218	30.4	16,274	38.3		5551	26.4	18,941	39.0	
CT or PET‐CT or Bone scan^a^	<3	7951	48.2	12,896	24.3	<0.0001	9903	36.6	10,944	25.7	<0.0001	6838	32.5	14,009	28.9	<0.0001
	≥3	8558	51.8	40,139	75.7		17,137	63.4	31,560	74.3		14,195	67.5	34,502	71.1	

^a^ Chi‐squared test.

^b^ Fisher's exact test

### 3.3 Survival according to pattern of imaging work‐up

Our analysis of the association between imaging follow‐up and clinical outcomes showed that the patients who underwent more systemic imaging follow‐up had higher rates of recurrence, breast cancer‐related mortality, and overall mortality (Table 4). To determine whether frequent imaging follow‐up resulted in a lower mortality rate, we separately compared the HRs for breast cancer‐related mortality and overall mortality according to the number of imaging studies performed for each modality (<3 vs. ≥3) within 3 years among patients who developed recurrence 3 years after surgery (Table 5). In univariate analyses, patients who underwent mammography ≥3 times showed a lower overall mortality rate (HR: 0.69, 95% CI: 0.59–0.81, *p *< 0.001) and breast cancer‐related mortality rate (HR: 0.69, 95% CI: 0.58–0.81, *p *< 0.001) compared to those who underwent mammography <3 times. After adjusting for age at diagnosis, chemotherapy, radiotherapy, adjuvant hormonal therapy, and comorbidities, only frequent mammography significantly influenced overall mortality (HR: 0.72, 95% CI: 0.61–0.84, *p *< 0.001) and breast cancer‐related mortality (HR: 0.72, 95% CI: 0.61–0.84, *p *< 0.001). After additionally adjusting for the stage at diagnosis, any breast imaging including mammography and systemic imaging did not influence survivals (Table 5).

**TABLE 4:** Univariable and multivariable Cox proportional hazard models for clinical outcomes by type of follow‐up frequency and modality

		Total (*N* = 69,544)	Death				Breast cancer‐related death				Recurrence			
			Event (*N* = 5241)	HR (95% CI)	aHR^a^ (95% CI)	aHR^b^ (95% CI)	Event (*N* = 4357)	HR (95% CI)	aHR^a^ (95% CI)	aHR^b^ (95% CI)	Event (*N* = 11,676)	HR (95% CI)	aHR^a^ (95% CI)	aHR^b^ (95% CI)
Mammography	<3	31,518	3179	1	1	1	2551	1	1	1	4944	1	1	1
	≥3	38,026	2062	0.53 (0.50–0.56)	0.55 (0.52–0.58)	0.53 (0.49–0.58)	1806	0.56 (0.54–0.61)	0.59 (0.55–0.62)	0.59 (0.53–0.65)	6732	1.12 (1.08–1.16)	1.08 (1.04–1.12)	1.14 (1.08–1.21)
Breast MRI	No	67,267	5163	1	1	1	4284	1	1	1	11,311	1	1	1
	Yes	2277	78	0.60 (0.48–0.75)	0.54 (0.43–0.67)	0.66 (0.51–0.87)	73	0.66 (0.53–0.84)	0.55 (0.44–0.70)	0.71 (0.54–0.93)	365	1.17 (1.06–1.30)	1.06 (0.95–1.18)	1.27 (1.13–1.43)
Chest radiography	<3	23,332	1269	1	1	1	905	1	1	1	3001	1	1	1
	≥3	46,212	3972	1.54 (1.45–1.64)	1.59 (1.49–1.70)	1.93 (1.74–2.15)	3452	1.89 (1.75–2.03)	1.86 (1.73–2.00)	2.35 (2.07–2.67)	8675	1.49 (1.43–1.56)	1.42 (1.37–1.49)	1.54 (1.45–1.63)
Chest CT	<3	58,270	3714	1	1	1	2932	1	1	1	8915	1	1	1
	≥3	11,274	1527	2.60 (2.45–2.76)	2.32 (2.18–2.47)	2.39 (2.18–2.61)	1425	3.04 (2.85–3.24)	2.58 (2.42–2.75)	2.75 (2.49–3.03)	2761	1.95 (1.87–2.04)	1.79 (1.71–1.87)	1.98 (1.87–2.09)
Bone scan	<3	37,825	2801	1	1	1	2171	1	1	1	5558	1	1	1
	≥3	31,719	2440	1.00 (0.95–1.06)	0.97 (0.92–1.03)	0.89 (0.82–0.98)	2186	1.16 (1.09–1.23)	1.08 (1.01–1.15)	1.04 (0.94–1.14)	6118	1.30 (1.26–1.35)	1.22 (1.18–1.27)	1.25 (1.18–1.32)
PET‐CT	<3	63,617	4560	1	1	1	3711	1	1	1	9977	1	1	1
	≥3	5927	681	2.16 (1.99–2.34)	1.79 (1.65–1.95)	2.49 (2.26–2.75)	646	2.47 (2.27–2.68)	1.96 (1.80–2.13)	2.83 (2.55–3.14)	1699	2.43 (2.31–2.56)	2.19 (2.08–2.31)	2.96 (2.78–3.14)
DEXA	No	45,052	4303	1	1	1	3601	1	1	1	8705	1	1	1
	Yes	24,492	938	0.47 (0.44–0.50)	0.45 (0.42–0.48)	0.51 (0.46–0.57)	756	0.45 (0.41–0.41)	0.43 (0.40–047)	0.49 (0.44–0.55)	2971	0.64 (0.64–0.69)	0.60 (0.58–0.63)	0.64 (0.60–0.68)
CT, PET‐CT, or bone scan	<3	20,847	1361	1	1	1	955	1	1	1	2858	1	1	1
	≥3	48,697	3880	1.35 (1.27–1.44)	1.26 (1.18–1.34)	1.40 (1.25–1.57)	3402	1.67 (1.56–1.80)	1.46 (1.36–1.57)	1.87 (1.63–2.16)	8818	1.44 (1.38–1.51)	1.30 (1.24–1.35)	1.51 (1.41–1.62)

^a^ Adjusted for age at diagnosis, treatment status (chemotherapy, radiotherapy, adjuvant hormonal therapy), and Charlson comorbidity index.

^b^ Adjusted for stage, age at diagnosis, treatment status (chemotherapy, radiotherapy, adjuvant hormonal therapy), and Charlson comorbidity index.

**TABLE 5:** Univariable and multivariable analyses for clinical outcomes by type of follow‐up frequency and modality in patients who developed recurrence 3 years after surgery

		Death (*N* = 4937)	Death				Breast Cancer‐related Death			
			Event (*N* = 616)	HR (95% CI)	aHR^a^ (95% CI)	aHR^b^ (95% CI)	Event (*N* = 575)	HR (95% CI)	aHR^a^ (95% CI)	aHR^b^ (95% CI)
Mammography	<3	2157	327	1	1	1	306	1	1	1
	≥3	2780	289	0.69 (0.59–0.81)	0.72 (0.61–0.84)	0.94 (0.65–1.35)	269	0.69 (0.58–0.81)	0.72 (0.61–0.84)	1.00 (0.69–1.45)
Breast MRI	No	4858	611	1	1	1	570	1	1	1
	Yes	79	5	0.82 (0.34–1.97)	0.61 (0.25–1.48)	0.21 (0.03–1.51)	5	0.87 (0.36–2.10)	0.63 (0.26–1.53)	0.22 (0.03–1.57)
Chest radiography	<3	1430	180	1	1	1	168	1	1	1
	≥3	3507	436	0.96 (0.81–1.14)	0.96 (0.80–1.14)	1.39 (0.93–2.06)	407	0.96 (0.80–1.15)	0.95 (0.80–1.14)	1.36 (0.91–2.04)
Chest CT	<3	4342	546	1	1	1	509	1	1	1
	≥3	595	70	1.28 (1.00–1.65)	1.15 (0.90–1.48)	1.00 (0.67–1.67)	66	1.295 (1.00–1.68)	1.14 (0.88–1.49)	1.07 (0.68–1.68)
Bone scan	<3	2532	327	1	1	1	304	1	1	1
	≥3	2405	289	0.90 (0.77–1.06)	0.88 (0.75–1.03)	1.04 (0.73–1.50)	271	0.91 (0.77–1.07)	0.84 (0.74–1.03)	1.09 (0.76–1.58)
PET‐CT	<3	4714	601	1	1	1	560	1	1	1
	≥3	223	15	0.96 (0.57–1.60)	0.74 (0.44–1.23)	0.94 (0.53–1.68)	15	1.02 (0.61–1.71)	0.77 (0.46–1.28)	0.98 (0.55–1.76)
DEXA	No	3508	486	1	1	1	452	1	1	1
	Yes	1429	130	0.83 (0.69–1.01)	0.88 (0.72–1.07)	0.86 (0.59–1.26)	123	0.85 (0.69–1.03)	0.90 (0.74–1.11)	0.88 (0.60–1.30)
CT, PET‐CT, or bone scan	<3	1553	218	1	1	1	202	1	1	1
	≥3	3384	398	0.92 (0.78–1.09)	0.86 (0.73–1.02)	1.17 (0.75–1.81)	373	0.93 (0.79–1.11)	0.85 (0.71–1.01)	1.21 (0.77–1.91)

^a^ Adjusted for age at diagnosis, treatment status (chemotherapy, radiotherapy, and adjuvant hormonal therapy), and Charlson comorbidity index.

^b^ Adjusted for stage, age at diagnosis, treatment status (chemotherapy, radiotherapy, adjuvant hormonal therapy), and Charlson comorbidity index.

## 4 DISCUSSION

This study found that among the imaging modalities used for follow‐up surveillance after curative surgery for breast cancer, only frequent mammography is associated with survival, and frequent use of other imaging modalities did not lower the rates of overall and breast cancer‐related mortality, particularly in patients who developed recurrence after surgery. Further, the pattern of imaging follow‐up differed by age, stage, and type of treatment.

Recent guidelines, such as those from the American Society of Clinical Oncology, National Comprehensive Cancer Network (NCCN), and European Society for Medical Oncology (ESMO) do not recommend other laboratory tests (e.g., tumor markers) or imaging tests (e.g., bone scans, chest or abdominal CT, PET‐CT) in asymptomatic patients because there is no evidence to support their survival benefit.^5^, ^18^, ^19^ However, these tests are being performed in clinical practice because of patient and clinician fear of recurrence or metastasis and the belief that early detection using more intensive imaging work‐ups reduces cancer‐related death. This is supported by the results of the current study, in which 97.2% of the patients underwent a systemic imaging work‐up that included a bone scan, CT, or PET‐CT. In a survey of clinicians on follow‐up after primary treatment of breast cancer conducted by the Korean Breast Cancer Society, most respondents indicated that they conducted more intensive follow‐up imaging work‐ups than recommended in the current guidelines,^11^ similar to the findings of the current study.

With respect to the patterns of imaging work‐ups, we found that they differed by age, stage, and type of treatment. In young women aged <30, more breast MRIs and fewer mammograms were performed. This could be because of the tendency for dense breast tissue and genetic susceptibility (BRCA1/2 mutation) in these patients.^20^, ^21^

Meanwhile, in patients treated with adjuvant hormonal therapy (i.e., those with hormone receptor‐positive breast cancer), more bone scans and fewer chest CTs were performed. This result could be interpreted to mean that clinicians tend to recommend different systemic imaging modalities according to the tumor characteristics, as hormone receptor‐positive tumors develop more bone metastases than visceral metastases.

We also found that systemic imaging work‐ups were more frequently performed in patients with advanced cancer and in patients who receive chemotherapy. However, this did not improve overall survival or breast cancer‐related survival. Previous randomized controlled trials have found that less‐intensive follow‐up strategies did not negatively affect patient outcomes or early detection of recurrence. In addition, more intensive follow‐up was associated with higher costs without differences in early detection of relapses.^22^, ^23^ In other systematic review article evaluating the clinical effects of intensive versus less‐intensive follow‐up on disease outcomes, intensive follow‐up with more frequent work‐ups did not reduce mortality and recurrences in breast cancer patients. ^24^ In addition, there was no survival benefit associated with the early diagnosis of recurrence by intensive follow‐up prior to the occurrence of symptoms, supporting the validity of the current guidelines.^9^ Moreover, one study reported that the intensity of imaging work‐up did not affect QoL in breast cancer survivors, and 70% of the patients even reported feeling more stressed and anxious when they visited clinics, especially after undergoing tests. ^25^ Expert panel on breast imaging according to ACR appropriateness criteria comment that there is no role for imaging to screen for distant recurrences in asymptomatic patients with a history of stage I breast cancer that received treatment for curative intent. They recommend that routine surveillance with an annual mammogram is the only imaging test that should be performed to detect an in‐breast recurrence or a new primary breast cancer in women with a history of stage I breast cancer. ^26^

This study has some limitations. First, the merged data used for the study did not include detailed information on tumor characteristics, such as hormone receptor status. To compensate, we analyzed the outcomes by adjusting for hormonal therapy status. Second, we were not able to take into account the indication for the imaging work‐ups such as cancer‐related symptoms, comorbidity‐related causes, or other medical issues or who prescribed the work‐ups (e.g., oncologists or primary physician) in our analyses. However, we considered the presence of morbid disease and the cause of death in our analysis of the effect of intensive imaging work‐ups on survival. Third, this study was not randomized or a planned prospective study. However, the national health insurance data and statistics which our analyses are based on are highly specific because the KNHIS is the only public health insurer and covers at least 98% of Koreans and is the only representative national database to include cause of death. ^27^

In conclusion, this study showed that as a follow‐up imaging modality, only frequent mammography and no other imaging modalities reduce overall mortality and breast cancer‐related mortality in Korean female breast cancer patients. These findings provide evidence that frequent systemic imaging work‐ups are not needed despite the fear of recurrence. Therefore, clinicians need to adhere to the current guidelines for surveillance after curative treatment in breast cancer patients.

## CONFLICT OF INTEREST

The authors declare no potential conflicts of interest.

## Data Availability

The data that support the findings of this study are available from the corresponding author upon reasonable request with the permission of KCCR, Statistics Korea, and KNHIS.
